# Adaptive and non-adaptive models of depression: A comparison using register data on antidepressant medication during divorce

**DOI:** 10.1371/journal.pone.0179495

**Published:** 2017-06-14

**Authors:** Tom Rosenström, Tim W. Fawcett, Andrew D. Higginson, Niina Metsä-Simola, Edward H. Hagen, Alasdair I. Houston, Pekka Martikainen

**Affiliations:** 1 School of Biological Sciences, University of Bristol, Bristol, United Kingdom; 2 Department of Psychology and Logopedics, Faculty of Medicine, University of Helsinki, Helsinki, Finland; 3 Centre for Research in Animal Behaviour, University of Exeter, Exeter, United Kingdom; 4 Population Research Unit, Department of Social Research, University of Helsinki, Helsinki, Finland; 5 Department of Anthropology, Washington State University, Vancouver, Washington, United States of America; Peking University, CHINA

## Abstract

Divorce is associated with an increased probability of a depressive episode, but the causation of events remains unclear. Adaptive models of depression propose that depression is a social strategy in part, whereas non-adaptive models tend to propose a diathesis-stress mechanism. We compare an adaptive evolutionary model of depression to three alternative non-adaptive models with respect to their ability to explain the temporal pattern of depression around the time of divorce. Register-based data (304,112 individuals drawn from a random sample of 11% of Finnish people) on antidepressant purchases is used as a proxy for depression. This proxy affords an unprecedented temporal resolution (a 3-monthly prevalence estimates over 10 years) without any bias from non-compliance, and it can be linked with underlying episodes via a statistical model. The *evolutionary-adaptation* model (all time periods with risk of divorce are depressogenic) was the best quantitative description of the data. The non-adaptive *stress-relief* model (period before divorce is depressogenic and period afterwards is not) provided the second best quantitative description of the data. The *peak-stress* model (periods before and after divorce can be depressogenic) fit the data less well, and the *stress-induction* model (period following divorce is depressogenic and the preceding period is not) did not fit the data at all. The evolutionary model was the most detailed mechanistic description of the divorce-depression link among the models, and the best fit in terms of predicted curvature; thus, it offers most rigorous hypotheses for further study. The stress-relief model also fit very well and was the best model in a sensitivity analysis, encouraging development of more mechanistic models for that hypothesis.

## Introduction

Major depression (MD) is characterized by symptoms such as prolonged anhedonia, low mood, and suicidality. The prevalence of MD is high in most age groups, including young and otherwise healthy adults (across all ages, ~14.6% experience it and ~5.5% had it less than year ago), and is responsible for a significant fraction of global disease burden [1,2]. MD episodes rarely occur without an associated exposure to a stressful life event [3–5]. Divorce and marital problem feature strongly among the potentially depressogenic events [5,6]. MD's responsiveness to divorce and other stressful life events has provoked two overarching classes of explanatory models: mainstream *diathesis-stress* models suggest that the psychological stress associated with the social problem provokes MD mainly in individuals that have some underlying vulnerability for it (MD as a ‘disorder’), whereas *adaptationist* theories propose that depression has a social role that increased biological fitness despite the associated emotional and physiological costs (MD as ‘normal distress’). Because most stressful life events are unpredictable, and many are difficult to verify independently, it is challenging to study their relationship to MD over time [7].

To overcome difficulties with accurate life-event assessment, Metsä-Simola and Martikainen studied a unique Finnish dataset on the population prevalence of psychotropic medication use in relation to the occurrence of divorce [6]. Here we use this same dataset to compare the quantitative fit of three alternative non-adaptive diathesis-stress models and an adaptive evolutionary model to patterns of depression during divorce. Due to the characteristics of the Finnish population registry (see Methods), the large and nationally representative Finnish sample allows the prevalence of antidepressant use to be estimated with high precision. It also affords an unprecedented temporal resolution of one prevalence estimate every three months for 10 years with no bias from attrition or repeated interactions with researchers (these data also have limitations that we discuss below). In this study, we take further advantage of this temporal resolution to help discriminate among different diathesis-stress and evolutionary theories of depression by fitting formal mathematical models (outlined below) to the Finnish data (these data also have limitations that we discuss below).

Divorce involves numerous practical and emotional upheavals, including new financial burdens, changes to social networks, parenting challenges, revisions to self-identity, and grieving the end of the marriage. Although most adults manage these upheavals well, some suffer MD and other mental health challenges [8]. An important and still unresolved question is whether poor mental health predisposes people to divorce (social selection) or the stress of divorce causes depression (social causation). Previous studies of the relationship between divorce and depression had found evidence for the roles of both selection and causation (for review, see previous studies [6,8]).

Metsä-Simola and Martikainen [6] classified all psycholeptics and psychoanaleptics into three groups: (1) antidepressants, (2) anxiolytics, sedatives, and hypnotics, and (3) other psychotropic medication (mostly antipsychotics). They then found that, compared to the continuously married, the divorced sharply increased their antidepressant use in the 18 months before divorce, with usage peaking shortly before the divorce was finalized, and then rapidly decreased their use for about 18 months after divorce. In contrast, there was a much smaller peak in the use of anxiolytics, hypnotics, and sedatives, and no increase in use of antipsychotics and other psychotropics. Moreover, use of both of the latter groups of psychotropics was elevated among the divorced compared to continuously married for five years prior to the divorce, indicating the role of selection (mental-health problems leading to divorce) among the users of psychotrophics other than antidepressants. There was also some evidence of elevated use of antidepressants among the divorced as early as five years prior to the divorce, especially among women. Nevertheless, the role of selection appeared strongest in case of mental disorders requiring treatment with antipsychotics, and the causal role of divorce on psychotropic drug use appeared strongest in the case of antidepressants. Sociodemographic variables had little effect on these trajectories. In addition to antidepressants differing from other psychotrophics in their relationship with divorce, depression differs from other psychiatric disorders in selective responses, or its association with fecundity [9].

Metsä-Simola and Martikainen proposed two possible explanations for the sharp rise and fall of antidepressant use in the 18 months before and after divorce [6]. The first is that this pattern reflects the prevalence of affective illness in proximity to a stressful life event. Many theories of depression are variations of the diathesis-stress model, wherein individuals with a dispositional vulnerability succumb to an episode of MD after exposure to a stressful life event [10,11]. In recent decades, research programs tended to focus on diatheses, such as susceptibility alleles [12], dysfunctional cognitions [13], personality traits [14–16], interaction styles [17], and ontogenetic factors [18]. Greater attention is now being paid to stress, especially the putative dysfunctions of the underlying biological mechanisms of stress responses and stress-linked genetic and epigenetic factors; the impact of early life factors, such as child abuse, on stress responses, and the distinction between acute and chronic stress exposure, are also receiving increased attention (for review, see Hammen [11]).

The second possible explanation is that the distress associated with divorce and other adverse life events is a natural response that does not indicate psychological dysfunction. From antiquity until the modern era, clinicians refrained from diagnosing illness when sadness or melancholy occurred in response to adversity, reserving the illness label for sadness in the absence of adversity, or sadness whose severity was disproportionate to the degree of adversity [19].

Horwitz and Wakefield [19] noted that the Feighner criteria and Research Diagnostic Criteria for depression, the basis for diagnosis of MD in DSM-III to DSM-V, relied on symptoms only and ignored social context (e.g., adversity). They argued that although these criteria were effective in fulfilling their original purpose–discriminating depression from other psychiatric disturbance in patient populations–by (mis)applying them to community populations, normal sadness and low mood were frequently mistaken for mental disorders, producing an extremely large rate of false positives (the high false positive rate was only partially ameliorated by the introduction of the “clinical significance” criterion in DSM-IV). Metsä-Simola and Martikainen thus raise the possibility that because the use of psychotropic medication has expanded, the trajectory of antidepressant use mostly reflects normal distress, not disorder [6].

The distinction between “normal distress” and disorder, however, and health and illness more generally, has been the subject of much debate. Wakefield [20,21] introduced the influential “harmful dysfunction” concept of illness, which is grounded in evolutionary biology. Illnesses, mental or otherwise, are (1) dysfunctions of biologically evolved mechanisms that (2) harm the individual. Physiological and psychological traits that are functional–that is, whose existence is explained by the fact that they increased the reproductive success of individuals possessing the trait *versus* those that did not possess the trait–are not illnesses even if they inflict high costs (we refer to such increased and decreased mean reproductive success over evolutionary time as fitness benefits and fitness costs, respectively, and to the functional traits as *adaptive* traits). The immune system is an example of adaptation that involves clear costs besides its benefits: it is energetically costly, releases highly reactive compounds that damage healthy tissue, and can accidentally activate in the absence of a pathogen, causing autoimmune diseases. Yet immunity is adaptive, not an illness, because the fitness benefits of killing pathogens outweigh all these fitness costs.

Sadness, low mood, and depression involve a number of fitness costs, including loss of interest in previously enjoyable activities, loss of appetite, and fatigue, all of which reduce productivity. When these costs are experienced for less than two weeks they are widely regarded as “normal.” When they are experienced continuously for more than two weeks they are widely regarded as evidence of dysfunction, and hence psychopathological MD. The conclusion that MD is pathological is seemingly strengthened by the fact that it is associated with increased mortality, partly due to a sharply increased risk of suicide [22–27]. Time spent in MD episodes appears to be a stronger predictor of the patients’ suicidal behaviours than other known correlates, and for example, the higher risk of suicidal behaviour associated with certain personality characteristics is mainly mediated by increased time spent in MD episodes rather than factors unrelated to MD [28–30]. While researchers have been interested in the possibility that adverse health behaviours, like smoking and alcohol use, might cause MD, these hypotheses are not supported by recent causality analyses [31–33]. While smoking appears to be causally associated with psychosis and use of antipsychotic medication, it does not cause depression or the use of antidepressant medication [31,33]. Therefore, MD is also a likely antecedent of mortality due to some physical causes. Yet, in contrast to other psychiatric disorders, the consequences of the depression-associated adversity on overall fecundity appear to be balanced by some unknown factors [9].

If there were no fitness benefits to compensate for the costs of any degree of sadness or low mood, these would simply be illnesses of varying degrees of severity. Many evolutionary theories involving different mechanisms of fitness benefit have been proposed, however; for review, see Hagen [34] and Durisko et al. [35]. Some theories propose that sadness and low mood improve decision-making under adversity, by, for example, focusing attention on, learning about, and avoiding threats [36]; letting go of unreachable goals [37]; reducing risk taking [38]; and seeking resolutions to social dilemmas [39]. Other theories propose that some symptoms of sadness and low mood are social strategies. Darwin [40] and Bowlby [41], for example, argued that sadness and low mood have deep evolutionary roots in infant cries that function to maintain proximity to the mother. Others have argued that, when adversity strikes, sadness, low mood, depression, and suicidality in adults function to credibly signal need to social partners and to bargain over resources [42–47] Still others propose that depression facilitates submission to dominant individuals [48], or that sadness and low mood provide a physiological benefit, such as saving energy when resources are constrained, or activating the immune system when social conflicts increase the risk of physical injury [49]. Many of these potential benefits are complimentary, and some theories unify learning, decision-making, social signalling and bargaining functions (e.g. [42,43,45]).

Most proponents of these various theories agree that the putative benefits outweigh only low costs of mild sadness, and that MD, as currently defined in the DSM and ICD, is pathologically severe. Some, though, argue that even particularly costly symptoms and behaviours, such as prolonged loss of interest in virtually all activities and suicide attempts, can yield a net benefit in situations of extreme adversity [34]. In addition to adaptive theories of MD, an adaptive origin has been recently suggested for a personality disorder that is highly comorbid with MD [30,50–52] and entails both interpersonal problems in the area of romantic relationships and use of costly bargaining strategies for mate retention [53].

### The current study

Metsä-Simola and Martikainen [6], based on a purely statistical analysis of the Finnish data, put forward qualitative verbal interpretations of the temporal patterns they found. Here we reanalyse the Finnish data using a quantitative approach that is common in many disciplines, such as physics, evolutionary biology, and infectious disease epidemiology, but is rare or non-existent in psychiatric epidemiology. We first develop mathematical models of four competing causal, mechanistic theories of the relationship between divorce and MD. We then use these models to generate four quantitative predicted temporal distributions of antidepressant use relative to divorce in the Finnish population. Finally, we quantitatively assess how well each predicted distribution fits the empirical distribution, which will allow us to rank the scientific plausibility of each mathematical model.

We fit and compare three diathesis-stress models (MD as disorder) and one adaptive model (MD as normal reaction) of depression during divorce (these are described in detail in the Methods section and S1 Text). There are three diathesis-stress models because divorce involves many aspects that could potentially cause psychological stress and those aspects distribute differentially with respect to the time of divorce. The first diathesis-stress model, which we term the *stress-relief* model, posits that the depressogenic stressors arise from pre-divorce adversity, such as marital conflict and efforts to save or terminate the marriage. The stressors terminate with divorce and thus so does reactive MD. That is, divorce signals the end of a chronic or prolonged stressor (“stress exposure”) and MD results from “stress responses” [7]. The second diathesis-stress model, which we term the *stress-induction* model, posits that the depressogenic stressors are caused by divorce, such as the loss of economic and other benefits, loneliness, shifts in childcare responsibilities, and negative impacts on children; the highest risk for MD then follows divorce rather than precedes it. That is, divorce signals beginning of stress exposures. The third diathesis-stress model, which we term the *peak-stress* model, posits that depressogenic stressors occur before and after divorce, but peak about the time of divorce. In other words, divorce either is a stress exposure or signals the average location of acute stress exposures associated with it. In this model, we do not assume that the ‘true’ acute exposure(s) would be at the exact same time point in relation to registered divorce but rather there is a population-average time around which most people’s stress exposures are distributed.

The above non-adaptive models are compared with an adaptive evolutionary model. Divorce is an ideal test case for the adaptive theories of depression because loss of a mate could have a profound impact on biological fitness. According to evolutionary theories, depression could have a range of possible benefits to offset this cost, as discussed above. All these theories have a common cost-benefit structure: under the unavoidable constraint of marital discord, individuals may adopt a depressed state (entailing fitness costs) to obtain potential fitness benefits and thereby achieve the optimal net fitness pay-off. We therefore subsume these evolved function theories of depression under a single *adaptive* model. From the viewpoint of current fitness, successful resolutions to marital discord could include postponement of divorce, reconciliation with partner or immediate replacement of the lost relationship, all which result in negligible period without a partner. Regarding adaptive models, we use words like “adopting” or “choosing” of a “strategy” for narrative fluency, without implying conscious deliberation (*cf*. above discussion on immune response and infant cries).

Temporal precedence is one of the classic signs of causality, but it is potentially ambiguous [54]. If temporal structure is to be used to make inferences about possible adaptive coping mechanisms in the presence of divorce risk, this may sometimes require an explicit model and high temporal precision in the data. For example, even though MD often precedes the divorce, this does not necessarily mean that the MD episode *caused* the divorce: if MD is a social negotiation strategy of a kind, as suggested by some theories [34–49], the mere fact that such strategies are often needed when there is a severe marital conflict could explain why MD frequently precedes realised divorces; however, this does not mean that the outcome would necessarily have been any better without MD. Surely also crying is more frequent before divorce than it usually is, but one would not be tempted to make a causality argument based on this. To get proper causal information, one should adjust for or randomize the conditions preceding the divorce and observe if MD (or crying) leads to divorce independent of the marital-conflict condition (which *already* entails higher than usual risk of divorce). In practice, it is extremely difficult to do such controlled trials without rather unethical experiments. For this reason, the empirical evaluation of our target models requires a more fine-grained analytic approach. We chose to use state-dependent models and dynamic programming [55–57]. as outlined in the Methods section. This approach will provide an explicit explanation for why some individuals have more acute and others more prolonged depression risk before divorce.

### Antidepressant purchases as a proxy for depression

It is currently impossible to measure depression directly. All current methods of measuring depression, including diagnostic interviews and self-reports using a variety of different depression scales, involve trade-offs in validity, reliability, sensitivity, specificity, time, and cost [58–63]. When Danish adults were surveyed using the “Major Depression Inventory” as the gold standard, antidepressant purchases were a highly specific (specificity of 0.93–0.97 in men and 0.89–0.95 in women) but not very sensitive measure of MD (0.30–0.44 in men and 34–55 in women) [64].

It is well-known that antidepressants are sometimes prescribed for other conditions than MD [65], and some early studies that relied on self-reported reasons for use seemed to indicate that only a minority of antidepressant users were being treated for depression. More recent studies that could directly link doctors’ diagnoses with antidepressant prescriptions, however, have found that a majority of prescriptions are for depression [66–69], and prescriptions due to other causes are more common in elderly than in middle-aged adults [66]. Especially selective serotonin reuptake inhibitor (SSRI) antidepressants have been prescribed most often for MD and tricyclic antidepressants (TCA) least often. To further support use of antidepressant purchases as a proxy for depression, we conduct a sensitivity analysis by separately studying SSRIs and TCAs, under the hypothesis that SSRIs reflect depression better than TCAs.

In addition, divorce is a known correlate of MD and Metsä-Simola and Martikainen showed that mainly antidepressant use, and not the use of other psychotrophic medications, respond to divorce [6]. Second, we only model changes in the prevalence of purchases around the time of divorce, meaning that any conditions that affect purchases but are unrelated with divorce are inconsequential. Third, all our models include a sub-model linking underlying depressive reactions with subsequent antidepressant purchases (see Methods) and the compared substantive models only differ from each other with respect to treatment of the underlying MD, not the purchases process.

The final and more subtle argument in support of our purchases proxy pertains to ecological validity. Even the most widely applied definitions of MD are debated [19,58,70] and therefore registered consequences of MD have certain ecological appeal as its indicators. For example, Rosenström & Jokela [58] showed that the current DSM definition includes a sub-population that have no disability and therefore may represent false positive diagnoses (the symptoms they found unnecessary have not been present in many older definitions of MD [60]). The false positive cases are unlikely to seek consultation and purchase antidepressants, and despite its limitations, the purchases proxy may have higher ecological validity in this respect in comparison to a formal interview diagnosis. A prescription requires seeking medical help. Thus, one useful way to think about our analysis is to interpret it as a complementary viewpoint that concentrates on ultimate, registered population-level consequences of psychological distress, given that no depression biomarker is yet available [71]. It is also worth noting that recently introduced network theory of psychiatric disorders implies that comorbidity is an inherent property of mental disorders, and consequently, ‘pure’ measures of MD, if attainable, may lose part of the relevant phenomenon [61].

## Methods

### Dataset

The data we use to evaluate the explanatory models were from a nationally representative random sample of 11% of the Finnish population, drawn from administrative registers. The linkage of the registers was based on the unique personal identification numbers assigned to all citizens of Finland, and was approved by the Ethics Committee of Statistics Finland (permission TK-53-1519-09); see previous study [6]. We specifically studied those who had divorced between the years 1995 and 2003 (inclusive). Only individuals who were at least 18 years old (official adulthood in Finland) in 1995, and who were aged at least 25 (to avoid trailing the preceding drug-use pattern to childhood) and no more than 64 at the time of divorce if divorced, were included in the sample [6]. The final sample comprised 304,112 individuals, 23,956 of whom had divorced before the end of 2004.

In Finland, antidepressants can only be prescribed by medical doctors and can be purchased with a prescription only from a licensed pharmacy, which sends the prescription information to the Social Insurance Institution of Finland for reimbursement purposes. All persons residing in Finland are entitled to reimbursement for medical expenses. Up to three months of medication can be purchased at one time, and the type of medication and date of purchase are recorded, along with the personal identification number. The prescription register includes the date of purchase and the type of medication according to the World Health Organisation’s Anatomical Therapeutic Classification. Antidepressant purchase (group N06A) dates were recorded from years 1995 to 2007. Time was divided into three-month periods and centred around the time of first divorce for those who divorced; multiple drug purchases during the same three-month period for the same individual counted as a single purchase event. All available data from 60 months before to 60 months after the first divorce were used to compute estimates of divorce-centred prevalence.

**Fig 1:**
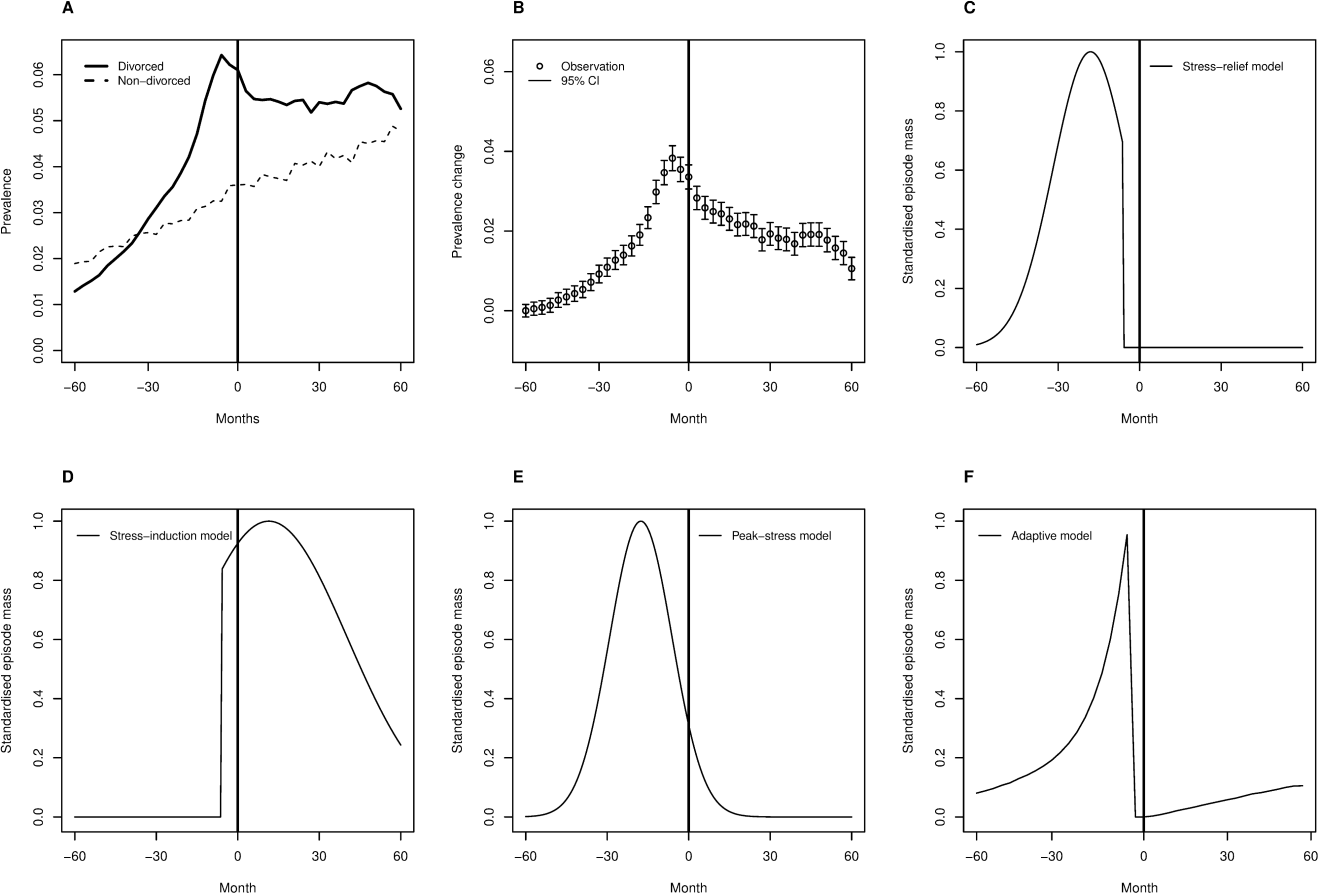
An illustration of data and explanatory models. (A) Prevalence of antidepressant purchases in divorced individuals in divorce-centred time (solid line) and in others in ordinary time (dashed line). The thick vertical line indicates the time of divorce. (B) The data points for model fitting and 95% Wald confidence intervals for prevalence; the general linear trend in non-divorced people and the baseline prevalence in divorced people was removed from prevalence data of the divorced individuals. (C) The stress-relief model (i.e. function *g*(*t*)) in the divorce-centred time t. (D) The stress-induction model. (E) The peak-stress model. (F) The adaptive model. Although all the model functions are shown here for illustration, their parametrisation is the one implied by the fitted models of the Results section.

The raw prevalence data on antidepressant purchases show a rising trend from 1995 to 2003 (as in the USA [65]), both for those who divorced in this period and for those who did not (Fig 1A). As we were only interested in the changes in prevalence of purchases due to the divorce process and not in overall trends of purchases, we removed the non-divorced group’s linear time trend from the divorced group’s data points and subtracted the divorced group’s minimum prevalence from all the data points. The models were evaluated against the resulting 41 preprocessed data points, shown in Fig 1B.

#### Data availability statement

The prevalence data to which we fit our models are not confidential; they are provided in the S1 Dataset. Access to national health registry is restricted by data protection laws and regulations concerning the national register-holding institutions (e.g., Finnish Law on Statistics, 2004/280 § 13). We are not allowed to make the data available to third parties. Interested researchers have the possibility to obtain data access by contacting the register-holding public institutions directly.

### Notation

Regarding our general notation, with function *g* we model population-level temporal changes in depressive status, whereas with function *h* we capture the overall rate of recorded purchases given the status. The function *h* and its parameters are introduced for the sole purpose of comparing different alternatives for *g* against each other, and are of marginal interest by themselves. In other words, the link between divorce and MD episode is assigned to *g* functions, whereas *h* captures the further link between MD episode and its proxy, purchase of antidepressants. More specifically, *g* is standardised and describes only the temporal changes in prevalence as a function of “divorce-centred” time (*t*) in months, with *t* = 0 signifying the time of divorce, and max_*t*_{*g*(*t*)} = 1. We first present the alternative theoretical predictions and their respective *g* functions (distinguished by subscripts *g*_*i*_, where *i* runs through 1, …, 4), then the *h* function common to all the predictions, and finally we explain how predictions of the models were compared against the data. Although the models are novel, their decomposition to *g* and *h* contributions is similar, for example, to convolution models in functional magnetic resonance imaging, where *g* would be a model of neural activity (the signal of interest) and the impulse-response function *h* would model the relationship between neural activity and its indirect, but measurable, blood-oxygen dependent signal [72].

### Diathesis-stress models

We compare three diathesis-stress models: the stress-relief model, the stress-induction model, and the peak-stress model. In the *stress-relief model*, we only want to assume that at some point before the registered divorce, the individual experiences depressogenic stress, which dissipates after divorce. Therefore, we take *g*_1_ to be a truncated normal distribution standardised to max_*t*_{*g*_1_(*t*)} = 1 and right-truncated at *t* = 0 (Fig 1C). We assume that the stress has a mode at time *μ*_1_ and dispersion *σ*_1_, and we estimate these parameters so that they yield the best fit to the data. The truncated normal was implemented using the R package “truncnorm”, version 1.0–7 (https://cran.r-project.org/package=truncnorm).

In Finland, there is a mandatory minimum consideration period of six months before a divorce is notified in the national register, and the modelled event (relationship breakdown) has typically occurred six months prior to a registered divorce. Thus, when we compare the stress-relief model against the data, we take the truncation at *t* = −6 months to registered divorce, such that the stressor is removed after the decision to divorce has been made (this choice also provided the best model fit).

In the *stress-induction model*, the divorce and associated separation and change of social status causes the depressogenic psychological stress. Therefore, *g*_2_ is based on a left-truncated normal distribution, instead of the right-truncation used in the stress-relief model. In all other aspects the model corresponds to the stress-relief model, but divorce-related depressive reactions concentrate on the time after divorcing rather than time before it (Fig 1D). Because of the mandatory consideration period, the truncation is made at −6 months when comparing the model with the data, so that the stressor begins after the divorce decision.

It is always possible in principle that materialisation of the divorce threat induces stress for some and relieves it for others, and that (possibly different) psychological stressors are associated with divorce before and after it. Therefore, we introduce the *peak-stress model*, where stress is normally distributed around some (estimated) mean and with some (estimated) variance; that is, *g*_3_ is based on the normal distribution, instead of the truncated normal (Fig 1E).

### The adaptive, state-dependent model

#### A model of evolutionary adaptive behaviour

In addition to capturing the alternative forms of diathesis-stress dynamics, we model the evolutionary adaptive dynamics of divorce and MD as follows. In our evolutionary model, stress does not have direct relevance to presence or absence of MD other than indicating the presence of adversity that requires adaptive behaviour, and perhaps by slightly increasing mortality risk associated with MD. What determines presence of MD is its effect on ultimate reproductive success.

A refinement of Lack's principle [73], as implemented in more complex life-history models of behavioural ecology [55–57] states that the fitness value of reproductive behaviours and behaviours affecting mortality risk is intimately related to the expected future events during the individual's remaining lifespan. To determine the net fitness outcome of a behavioural strategy (here MD as a response to divorce risk), one therefore needs to compute how its use at a given time point will affect the entire remaining lifespan. Note that MD may both reduce the probability of divorce and increase the probability of dying, with important consequences for future prediction; these effects need to be explicitly taken into account because natural selection operates on the net long-term fitness benefit rather than immediate outcomes.

Assume that a depressive reaction leads to a decrease *s* in the probability of divorce when a relationship is at risk e.g. through its bargaining leverage [43] or social-problem solving benefits [39], but increases probability of dying (mortality) by some amount *z*, as is known to happen [22–30]. Although materialisation of divorce on average reduces immediate reproductive opportunities, it does not preclude the possibility of finding a new partner later on with some uncertainty and delay. From the viewpoint of evolution, on one hand, selection works against organisms who waste reproductive opportunities because time (lifespan) is a limited resource, but on the other hand, such a waste can be tolerated if it implies more time (lifespan) to find new opportunities for reproduction. Thus, inferring the net fitness benefit of having MD now requires knowledge not only on the values of *s* and *z* but also on the remaining reproductive lifespan and the expected properties of the environment. We bring these aspects of evolutionary modelling together using the following abstract but quantitative, state-dependent model.

**Fig 2:**
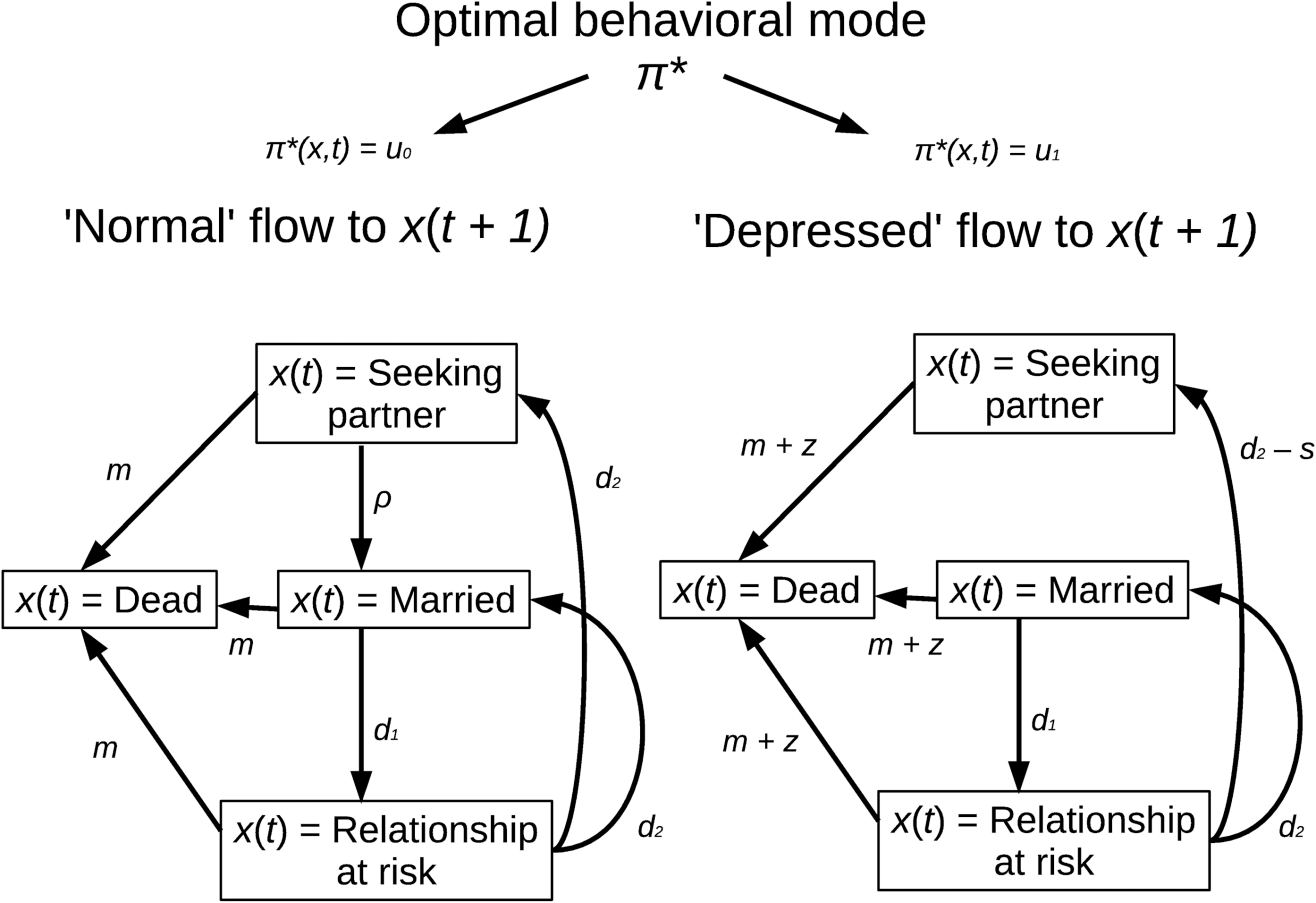
Possible state transitions in the evolutionary state-dependent model. Note that it is also possible to stay in the same state for longer than one time step, but for clarity the self-loops are not shown (but see S1 Text). A ‘strategy’ π(*x*,*t*) defines whether to be in a ‘depressed’ mode *u*_1_ or not (mode *u*_0_) given the state *x* and time *t*. The star superscript refers specifically to the optimal strategy that maximizes the reproductive value. The choice of mode dictates the transition-probability structure for the next time step. The effect of the ‘depressed’ mode is to decrease the probability of a divorce-like transition from the relationship-at-risk state to the unpartnered state by the value *s*, to increase the probability of death *m* by *z*, and to remove the probability of marrying *ρ* (removal of *ρ* had no consequences here, but to some, it seems a logical outcome of depression). We assumed that as many relationships at risk end up in divorce as in reconciliation on average (*d*_2_); the function *f*(*d*_1_, *d*_2_) captured the overall divorce rate, as illustrated in S1 Text.

We model a range of 20 years in 3-month periods and assume that, in each period *t* (where *t* refers to non-centred, ordinary time), individuals can occupy one of four exclusive states: *x*(*t*) = ‘seeking partner’; *x*(*t*) = ‘married’ (i.e. reproduction is possible); *x*(*t*) = ‘relationship at risk’ (reproduction is possible, but there is a chance that the relationship will terminate before the next period); and *x*(*t*) = ‘dead’ (no further reproduction). Between each period, individuals can transit along the arrows of Fig 2 with particular probabilities that depend on a chosen behavioural mode as detailed in the Fig 2: consciously or sub-consciously, individuals may ‘choose’ to be in a depressed mode *u*_1_ that subtracts *s* from the probability of divorce (i.e. transition from *x*(*t*) = ‘relationship at risk’ to *x*(*t*+1) = ‘seeking partner’) with the cost of adding *z* to the mortality risk, compared to the non-depressed mode *u*_0_.

Having set up a state-dependent model (details below), we use it to estimate *when*, if ever, it is evolutionarily adaptive to be “depressed” (in the mode *u*_1_). This is the behavioural strategy to which evolution is assumed to have converged {a strategy is a sequence of choices, or choice probabilities, given the environmental states and time, i.e. a function *u*(*t*, *x*) = Pr(*U* = *u*_1_ | *t*, *X = x*)}. We used dynamic programming [55–57]to identify the age- and state-dependent conditions under which the use of *u*_1_ maximizes an individual's reproductive value over the entire focal period (e.g. age 20 to 40 years) [56]. This ‘age’ period, used in simulations, aims to capture the general notion that reproductive lifespan is limited; we do not mean to imply that potential for depression would vanish after its reproductive role has been depleted any more than other state-dependent evolutionary adaptations (e.g., pain sensitivity or immune response). But as explained above, natural selection works on lifetime rather than immediate reproductive success, and evolutionary arguments about the existence of state-dependent behaviour need to be factored into the context of (at least) the entire reproductive period if they are to be understood (S1 Text provides a sensitivity analysis where all the relevant life course parameters are derived from modern-day, age-dependent statistics based on the studied population).

The following values of environmental parameters were used. State *x*(*t*) = ‘dead’ is absorbing (a zero probability of moving to other states at *t* + 1). Assuming that globally approximately one in eight people die between the ages 20 and 40 [74], and that time is divided into 3-month periods, the rough baseline probability of dying per transition in the usual behavioural mode can be computed as 1 –(1–1/8)^(1/81)^ ≈ 0.0016 (parameter *m* in Fig 2; here the same for all times *t* because predicted behaviour in the adaptive model depends mainly on depression-related trade-offs in mortality and fecundity rather than their baseline variations, but S1 Text provides an example of explicit age-dependence in *m*_*t*_).

Activating the depressed behavioural mode *u*_1_ adds *z* to the probability of dying. A high estimate of all-cause mortality was used as a starting point, because high costs set a conservative limit for inferring adaptive value from a model fit; this implied 3.1-fold mortality for men suffering from major depression and 1.7-fold for women [26]. Thus, *z* was set at 0.0024, representing a 2.5-fold increase in the hazard (this is a conservative limit because a 1.5-fold mortality increase has been often reported [24,25]). The depressed behavioural mode trades the increased mortality risk for a social leverage of strength *s*. The model predictions were found to be robust against large changes in *s* (S1 Text), but the value *s* = 0.007 offered nevertheless the best fit when exploring range of values. This value was used in analyses.

The probability of ‘marrying’ when in state *x* = ‘seeking partner’, *ρ*, was defined from the average annual hazard for entry into the first cohabiting or married relationship [75]. Because that average was estimated as ~0.028, the 3-monthly probability was taken to be *ρ* = 1 –(1–0.028)^1/4^ (explicit age-dependent marriage data was used in S1 Text). We assumed that there is zero probability for a new relationship in the depressed behavioural mode *u*_1_, although assuming the same probability for both modes, *u*_0_ and *u*_1_, made little difference to the model predictions. Fertility was constant in the model until vanishing in the last time period (any constant yields the same predictions; age-/time-dependent fertility was used in S1 Text) [56].

Finally, we needed to fix the transition probabilities for entering from ‘married’ state at time *t* into the ‘relationship at risk’ state at *t+*1 and then further to divorce {i.e., *x*(*t+*2) = ‘seeking partner’}. As this involves two transitions and empirical estimates exist only for divorce rates, there is ambiguity in possible transition probabilities; here, the annual divorce hazard was approximated as 0.025 [76], and a range of transition probabilities producing this hazard were explored as described in the S1 Text (the empirically observed annual hazard was achieved by setting both transition probabilities to 0.07). A version of the behavioural strategy with infrequent “choice errors” was used, because it provides many technical benefits (smooth objective functions) and is biologically reasonable [56,77]; that is, the mode *u*_1_ is chosen with a probability proportional to a fixed parameter *δ* controlling the error rate (here *δ* = 0.0005).

#### Comparing adaptive and empirically observed behaviours

After identifying the optimal strategy through dynamic programming, we simulated a population (N = 50 000) of optimally behaving (‘evolved’) individuals. Starting from an initial frequency distribution (at age 20, *t* = 0) across the four states that approximately matched the data (i.e., 0.97 for *x* = 1, 0.02 for *x* = 2, 0.01 for *x* = 3, 0 for *x* = 4) [78], the individuals were tracked through 80 subsequent 3-month time periods (to age 40, *t* = 80). All divorce events (transitions from *x* = ‘married’ to *x* = ‘seeking partner’) and their times of occurrence were recorded from the simulated data (as for the real data), excluding those events that occurred within 20 periods of the beginning or the end of total modelled interval. For those simulated individuals that divorced, time was centred around their age at their first divorce (giving new time *t'* = *t − t*_divorce_). The standardized prevalence of the depression-like behavioural mode *u*_1_ as a function of divorce-centred time periods *t'* forms the predictive function *g*_4_(*t'*) for the adaptive model, in analogy to *g* in the non-adaptive stress-relief (*g*_1_), stress-induction (*g*_2_) and peak-stress models (*g*_3_).

Because of the mandatory minimum consideration period of six months before a divorce is notified in the national register, the modelled event (relationship breakdown) has typically occurred six months prior to a registered divorce (or possibly before, but importantly for the present paper, the same estimate was used for all the compared models). As our modelled ‘decisions’ already take at least three months to realize, we needed to take into account the above legislative lag by introducing a further single time-point lag into the model prediction (i.e. a ‘leftward’ shift by three months) before comparing it with the data.

### Nuisance parameters and a model for antidepressant purchases

We model population-level temporal changes in depressive status with a model *g* that yields the divorce-related depressive reaction *g*(*t*) at time *t*. The data *y*(*t*) signify the trend-corrected rate of antidepressant purchases. Comparison of *g*(*t*) with *y*(*t*) requires a model for the proportion of depressive reactions that lead to purchases and for the period that the purchases continue once initiated. Some continuation use is known to occur as recommended in the national guidelines for the prescribing doctors: “antidepressant treatment should always be continued approximately for half a year after the acute phase” [79]. Some patients stop taking drugs based on their own decision rather than the doctor’s recommendation [79], but a dependence may develop too [80], which implies variable stopping times. To account for this we used an impulse-response function *h*(*t*; *λ*, *α*) = *αλe*^*-λt*^, where *αλ* is the proportionality constant and *e*^*-λt*^ the exponential decay in the probability of continuing use after MD has lifted.

To predict the drug-purchase behaviour as a function of modelled depressive episode, the impulse-response function *h*(*t*; *λ*, *α*) was convolved with the depression model’s prediction of depression prevalence in divorce-centred time, denoted by *g*(*t*). The resulting model, (*h* * *g*)(*t*; *λ*, *α*) was fitted to the data *y*(*t*) by minimising the sum of squared residuals with respect to *λ* and *α* as explained in the Model estimation section. Parameters of the purchases model correspond to a model element frequently referred to as “nuisance parameters”: their estimation is necessary but of no substantive interest, as the aim was to compare the models for *g* rather than study *h*. If the model of interest, *g*, had free parameters, they were estimated simultaneously with the nuisance parameters *λ* and *α* (note that the need to estimate the constant *α* means that the total model is equivalent for any max_*t*_{*g*(*t*)}, which is why max_*t*_{*g*(*t*)} = 1 standardization was used for the diathesis-stress models, and max_*t*_{*g*_4_(*t*)} already is close to 1; convolutions are discussed more in S1 Text).

### Model estimation and comparison

Notice that in contrast to the mode and dispersion parameters in the non-adaptive models, *g*_4_(*t*) for the adaptive model did not have *estimable* free parameters. All the parameters in Fig 2 were fixed on the basis of *previous* population studies (see above section on state-dependent, adaptive model). Therefore, the total model (purchases model * explanatory model) had only two estimable parameters in the case of the adaptive model, (*h* * *g*_4_)(*t*; *λ*, *α*), and four estimable parameters in the case of the non-adaptive models; (*h* * *g*_*i*_)(*t*; *λ*, *α*, *μ*, *σ*), when *i* = 1, 2, or 3. The parameters, collectively denoted by *θ*, were estimated by minimising the residual sum of squares,

$$RSS=\sum_{t}{\left\{y(t)-(h*g)(t;\theta )\right\}}^{2},$$

with respect to *θ*, using the “optim” general-purpose optimisation function in the statistical software package R (64-bit Linux-version 2.15.3; www.r-project.org); R was also used for all other computations (see S1 Code for an example of estimation of the evolutionary adaptive model using R). In all cases, the optimisation converged without problems with the “L-BFGS-B” algorithm of the “optim” function [81].

We compared the models in terms of the proportion of variance in the temporal changes of antidepressant purchases that they explained (Coefficient of Determination, *R*^2^). Since *R*^2^ does not take into account the different number of free parameters, we also used Akaike Information Criterion (AIC), defined as 2*k* + *n* ln(RSS/*n*), where *k* is the number of estimated parameters, *n* is the number observations, and ln denotes natural logarithm. Here, the observations were the prevalence data points of Fig 1B, and therefore *n* = 41; *k* is either 2 or 4 depending on the model. We also report the Bayesian Information Criterion (BIC), defined as *k* ln(*n)* + *n* ln(RSS/*n*). The model with the lowest BIC and AIC values has the best complexity-corrected fit, but BIC and AIC have slightly different properties. BIC is a statistically consistent model selection procedure (as sample size tends to infinity) but sometimes AIC performs better than BIC in small samples; in general BIC has greater emphasis on model parsimony than AIC (i.e., the fit penalty from extra parameters is *k*ln(*n)* in comparison to the 2*k* in AIC) [82,83].

For purpose of quantitative evaluation of observed curvature patterns, we also computed a sum of squared differences between model-predicted and empirical 2^nd^ order central finite differences. This is an approximation for the quantity ∫{*y*''(*t*)–(*h* * *g*)''(*t*)}^2^*dt* that we call “Curvature Mismatch” (CM). Because its second derivative is closely related to curvature of a graph, the integrated (or summed) squared difference in empirical and model predicted second temporal derivatives quantifies the degree of model failure in terms of curvature. Curvature of a temporal pattern is of special interest because the shape of temporal evolution reflects the underlying mechanistic process; regarding probability distributions, a Gaussian (normal) shape is typically associated either with error variation or amalgam (sum) of several underlying sources, whereas distinct isolated mechanisms tend to generate non-Gaussian distributions [84].

## Results

**Fig 3:**
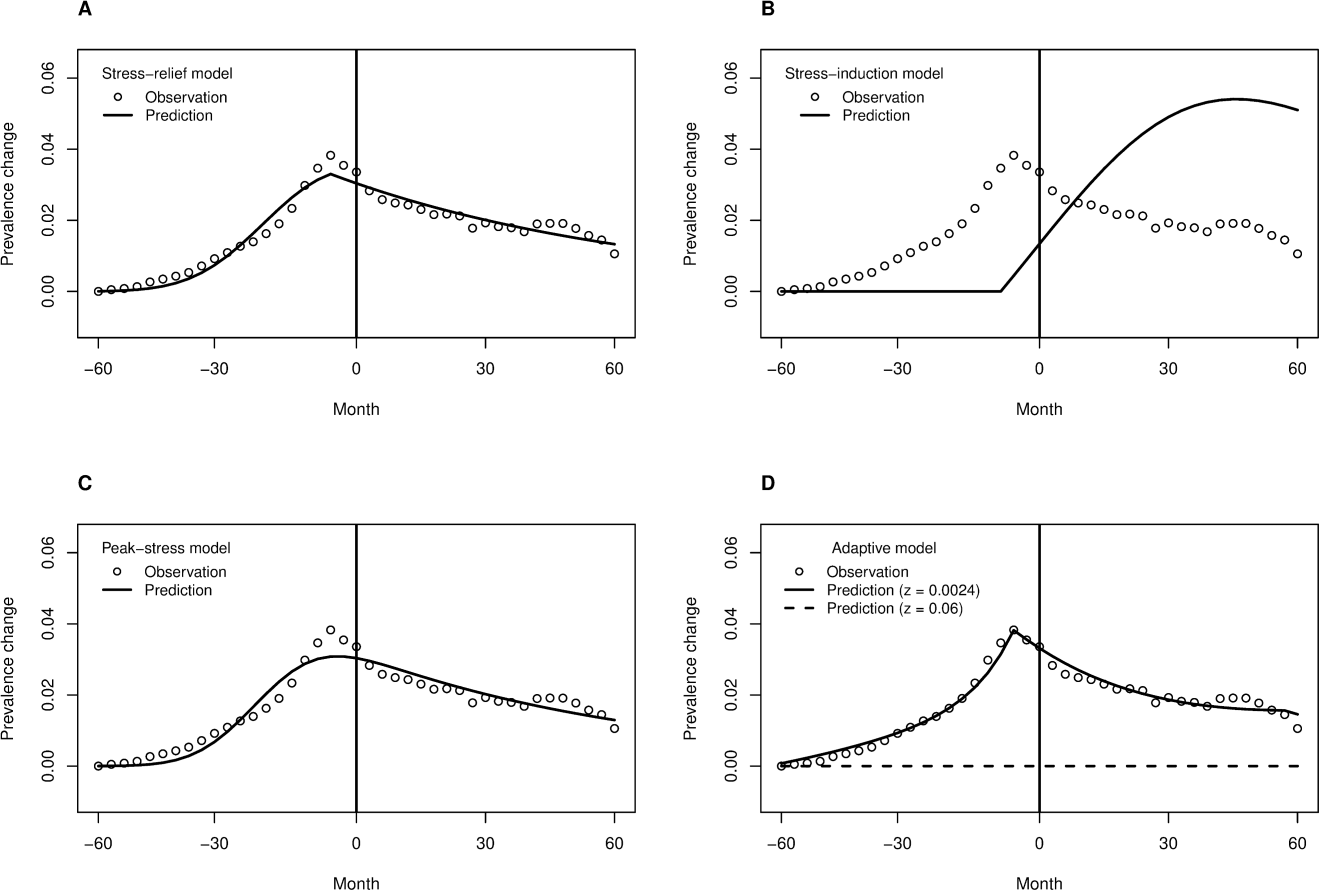
Model estimates compared with the data. (A) The stress-relief model. (B) The stress-induction model. (C) The peak-stress model. (D) The adaptive model. Solid lines show the model of interest, whereas the dashed line illustrates what would happen in the adaptive model if depressive episodes would imply roughly 15 times the mortality compared to the model of interest (*m* + *z*, with *z* = 0.06, rather than *m* + *z*, with *z* = 0.0024); in that case, the depressed mode of behaving would no longer be adaptive under any circumstances.

Fig 3 shows the fit of the estimated total models for each of the studied explanatory models, whereas the Table 1 displays the associated fit statistics. We discuss the findings for non-adaptive, diathesis-stress models first, then for the evolutionary adaptive model, and finally in terms of the overall model comparison.

**Table 1:** Parameter estimates and fit statistics for the models.

Model	*α*	*λ*	*μ*	*σ*	*k*	*R*^2^	AIC	BIC	CM
1. Stress-relief	0.1022	0.0414	-6.021	4.594	4	0.949	-340.18	-485.59	3.04∙10^−6^
2. Stress-induction	0.1140	0.0464	3.805	9.632	4	-4.512	-147.82	-293.82	4.61∙10^−6^
3. Peak-stress	0.0985	0.0447	-5.872	3.841	4	0.926	-324.65	-470.05	3.69∙10^−6^
4. Adaptive	0.1315	0.0709	-	-	2	0.972	-367.95	-516.78	2.97∙10^−6^

Note: *α*, *λ*, *μ*, and *σ* are model parameters, and *k* their total number

R^2^ = Coefficient of determination, or explained variance

AIC = Akaike's Information Criterion

BIC = Bayesian Information Criterion

CM = Curvature Mismatch

### Fit of the diathesis-stress models

Quantitatively, the stress-relief model fits the data very well. In psychology and psychiatry, it is not common to find models that explain 95% of the empirically observed variance. Qualitatively, however, one can observe that the curvature of the model prediction differs from that of the data-point sequence before the divorce (Fig 3A). This suggests that the model may be missing out something essential, despite its good numerical fit.

In contrast to the stress-relief model, the stress-induction model is a grossly inaccurate description of the data; because the model is nonlinear, this bad a fit can readily result in a negative *R*^2^ (as seen in Table 1). The negative *R*^2^ means that even the average of the data points is a better model for all the data than the stress-induction model. Thus, it is safe to conclude that the divorce itself does not predominantly cause *subsequent* psychological stress that would present as antidepressant purchases.

The peak-stress model fits to data quite well. Nevertheless, it has the same curvature miss-match compared to the empirical pattern as the stress-relief model, and in addition, it is worse at capturing the highest purchase prevalences just before the time of divorce (Fig 3C). Quantitatively, the peak-stress model is a good fit, though clearly worse than the stress-relief model. The BIC difference of ~15.5 would be generally considered “strong” evidence for the stress-relief model over the peak-stress model [83].

### Fit of the evolutionary adaptive model

The adaptive model provided the best numeric fit to the data (Table 1). In visual inspection, it also had almost the same curvature as the real data points (Fig 3D). The adaptive model makes the argument that fitness benefits can outweigh the fitness costs of depression. One of the results then is that even a comparatively (empirically) high mortality risk from *u*_1_ (i.e. 2.5-fold) does not outweigh the (theoretical) reproductive benefit from halving the probability of divorce just for the next 3 months. Of course, arbitrary risks could not be tolerated according to the model, as illustrated by the dashed line of the Fig 3D, which shows the prediction of the same model under 38.5-fold mortality due to the use of the depressed mode, *u*_1_ (see S1 Text for more information on potential trade-offs among the modelled environmental characteristics).

Because the efficacy of the adaptive behaviour, or value of the *s* parameter, had some effect to fit despite considerable robustness (S1 Text), it could be seen as a third parameter of the adaptive model. Nevertheless, the AIC (-365.95) and BIC (-513.06) values would compare favourable to the other models (Table 1).

### Comparison of the models

Both the (non-adaptive) stress-relief model and the adaptive model fit to the data very well according to the numerical fit criteria, with the adaptive model providing the overall best numerical fit. Upon visual inspection, the adaptive model captures the curvature profile of the data better than the stress-relief model. This observation also holds for our numeric assessment of curvature mismatch between the models and the data (Table 1). In all respects, there was a bit less support for the peak-stress model than for the two best models, and no support at all for the stress-induction model.

### Sensitivity analysis by type of antidepressants

**Fig 4:**
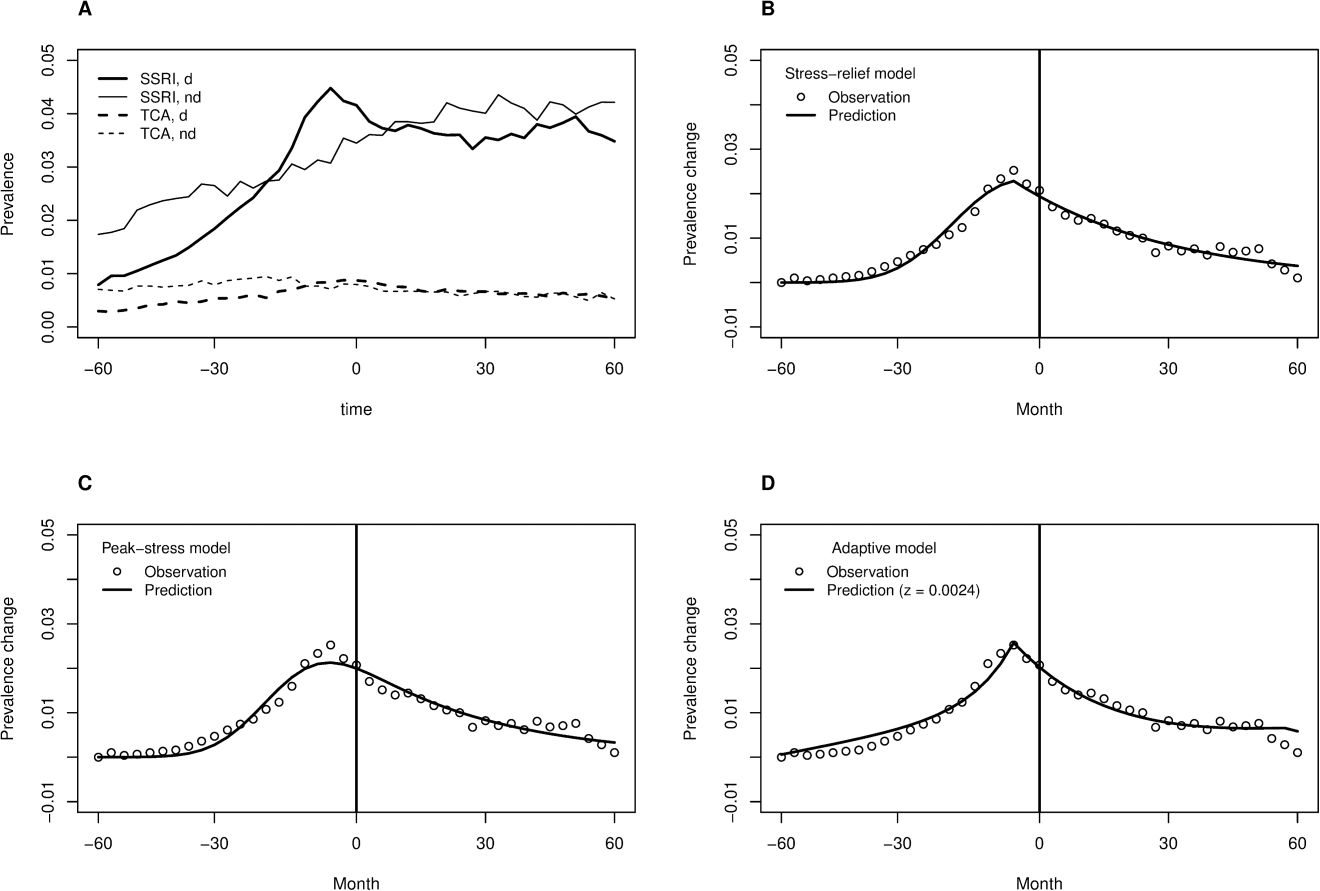
Sensitivity analysis using SSRI data. (A) Prevalence of selective serotonin reuptake inhibitor (SSRI; solid lines) and tricyclic antidepressants (TCA; dashed lines) purchases in divorced (d; thick lines) and non-divorced (nd; thin lines) individuals. Time is centred around the divorce for the divorcees. (B) Fit of the stress-relief model (line) to the SSRI data points (circles). (C) Fit of the peak-stress model. (D) Fit of the adaptive model.

Antidepressants are sometimes prescribed in other conditions than MD, and the frequency of non-MD indications varies between types of antidepressants. The available studies indicate that tricyclic antidepressants (TCA) are often prescribed for other reasons than MD, whereas selective serotonin re-uptake inhibitors (SSRI) are prescribed for MD in great majority of cases [66–69]. Panel A of Fig 4 shows that mainly SSRI prevalence corresponds closely to the above-studied pattern of antidepressant use, whereas TCA prevalence is low and does not show as clear peak for divorced (d) *versus* non-divorced (nd) individuals. This provides additional support for the idea that peak in antidepressant use around time of divorce reflects cases of MD rather than other non-MD conditions. Remaining panels of Fig 4 show model fit for three of the four models. The stress-induction model failed equally badly for SSRIs as it did for all antidepressant purchases and is therefore omitted from the Fig 4.

Performance of the adaptive model was more sensitive for the specific value of *s* parameter in case of SSRIs, and it was therefore considered as a 3-parameter model. Otherwise the results roughly resembled those for all antidepressant purchases, but the adaptive model (R^2^ = 0.939, AIC = -365.89, BIC = -513.01, CM = 3.41∙10^−6^) now fit slightly worse than the peak-stress model (R^2^ = 0.942, AIC = -365.83, BIC = -511.23, CM = 3.65∙10^−6^) and the stress-relief model (R^2^ = 0.959, AIC = -371.64, BIC = -525.04, CM = 3.24∙10^−6^), which was the best-fitting model.

## Discussion

We found that among the four explanatory models of the relationship between divorce and depression-biased psychiatric distress (assessed by antidepressant purchases), the evolutionary-adaptation model (all time periods with risk of divorce are depressogenic) provided the best quantitative description of the data, with the curvature of predicted prevalence around the time of divorce most closely resembling that of the observed data. The stress-relief model (period before divorce is depressogenic and period afterwards is not) provided the second best quantitative description of the data. The peak-stress model (periods before and after divorce can be depressogenic) fitted the data less well, and the stress-induction model (period following divorce is depressogenic and the preceding period is not) did not fit the data at all. None of these models were proposed as being exactly ‘true’ over the entire population, rather, their relative merits rank the dominant phenomena in the population (see S1 Text for discussion on sub-populations etc.). The model aims to synthetize research on depression, but like all models of depression, should be further studied in the light of known heterogeneity of depression and with respect to its alternative operationalisations.

In a sensitivity analysis using SSRI class of antidepressants only, the stress-relief model fit better than the other models. This was a slightly paradoxical outcome as SSRIs may be more specific to MD indication than other antidepressants [66–69], and thus the theory of the adaptive model should have been even better tailored for SSRIs than for all antidepressants. We do not read too much into this, however, as also the overall trends in antidepressant purchases of non-divorced individuals appear to get less clear when focusing on sub-types of antidepressants instead of all antidepressants (cf. Fig 4A *vs*. Fig 1A), and the differences in model fits were not big. In any case, the divorce-related pattern of antidepressant use was much more clear for the relatively MD-specific SSRIs in comparison to non-specific TCAs (Fig 4A). It is also more clear for antidepressants than for other types of psychotrophic medication [6]. This suggests that the divorce-related changes in antidepressant purchases were driven by MD instead of other mental disorders.

Both the stress-relief model and the adaptive model suggest that social conflicts preceding divorce are the likely cause for depressive episodes and subsequent antidepressant purchases. The adaptive model *explains* the distribution of episodes before the time of divorce, however, whereas the stress-relief model simply estimates it from the observed data. Specifically, given the population rates observed in other studies, assuming that depressive episodes help in solving the social conflicts that threaten marriages, and assuming that natural selection has optimised the use of such adaptive behaviour, the distribution of episodes around the time of divorce (Fig 1F) follows by mathematical deduction. For this reason, the adaptive model is a much stronger theoretical tool than the stress-relief model, provided that it is correct.

In addition to the quantitative results, our theoretical analysis is revealing in terms of the discussion on causality. For example, Bulloch et al. [85] found that MD did not precede transitions from unmarried to married status, but clearly did so for transitions from married to divorced status. They interpreted this to mean that MD increases marital discord. Whereas the finding is in line with the present findings, the evolutionary adaptive model revealed that it can also arise under the opposite causality wherein MD *decreases* risk of divorce in prevailing states of discord. Already Bradford Hill in his classic work on causality in epidemiology recognised that temporal precedence alone is often insufficient to establish causality [54]; our evolutionary model provides an example of this. Bulloch et al. [85] also reported weak evidence (p = 0.04) for a reverse association wherein divorce preceded later MD. Such a small effect would be consistent with our peak-stress model, but we found the model a sub-optimal fit, and definitely ruled out dominant temporal order from divorce to MD (the stress-induction model). Marital discord before divorce is likely to be prolonged stressor in comparison to the acute event of divorce, and chronic stressor have been found more strongly associated with depression than acute stressors [5,11]. Also a recent propensity score analysis showed that, after controlling for the propensity to separate, divorced individuals without MD were not more likely to get MD after the divorce than continuously married individuals without MD [8].

Although our model comparison procedure on the three diathesis-stress models appears to have been effective in reaching largely the same conclusions as the recent propensity-score causality analysis [8], it could be said that the three diathesis-stress models lack theoretical depth in comparison to the evolutionary adaptive model. The relative theoretical deficiencies of our stress models of MD could be addressed by formulating these models in terms of hypothesized dysfunctions of the underlying mechanisms. For example, one recent theoretical synthesis proposed that chronic stress dysregulates the mesocorticolimbic dopamine pathways that play key roles in reinforcement learning and motivation; dysregulated behaviour-reinforcement and motivation mechanisms, in turn, cause anhedonia, a cardinal symptom of MD [86]. There are well-developed mathematical models of reinforcement learning, such as the Rescorla & Wagner model [87], that appear to explain important aspects of dopamine signalling [88,89]. Hence, mathematical models of MD as stress-dysregulated learning and motivation could generate precise predictions of MD prevalence over time that could be compared to the predictions of our adaptive state-dependent model. In addition to dysregulation, also adaptive learning processes can lead to anhedonia-like inactivity in some cases [90].

The adaptive model might appear limited by its crude assumption of constant fertility, with a sudden change to zero fertility, and indeed we treat the topic at length in the S1 Text. However, precise fertility (or mortality) estimates may be less relevant for evolutionary adaptive arguments than they initially appear. The important things are the trade-offs between mortality risk, opportunities for reproduction and limited lifespan. These appear the most essential environmental (or life-history) constraints to which behavioural states should adapt. The specifics of the properties vary across evolutionary time, meaning that the parameters are a moving target in reality. Future studies could try to explicitly model this aspect too, but our S1 Text at least suggests that the model is quite robust to such variance.

Furthermore, it is possible that life-history constraints give rise to state-dependent behaviour under natural selection without the entire life course being optimized accordingly. Since life-history adaptations increasingly lack relevance for natural selection as reproduction decreases in old age, it is difficult to predict what happens to them after menopause based on evolutionary arguments only: they might tune out gradually, stay as they were, or became more active due to other age-related changes in body and external environment. For example, the immune system does not simply stop functioning after the menopause. Possible indirect fitness benefits from investing in children and grandchildren complicate the evolutionary analysis of specific adulthood age periods in humans.

### Limitations and suggestions for future research

Although Nordic population registries are widely used in psychiatric research and MD appears phenomenologically similar across nations [91–93], potential issues in cross-national generalizability should be kept in mind when interpreting the results. Furthermore, our antidepressant-based proxy only indicates presence or absence of MD, and it would make sense to further study levels of MD and possible heterogeneity between specific depressive symptoms [94]. In addition, antidepressants are often used to treat other disorders besides depression [65–69], and depressed individuals might seek out other treatments (e.g. psychotherapy) or not seek treatment at all. The prevalence of antidepressant use thus only approximates the prevalence of MD. However, our results rely primarily on the *change* in prevalence of antidepressant use, which probably better approximates the change in prevalence of MD than the absolute prevalence level. The trajectory of antidepressant use seen in our results is also similar to the trajectory of psychological distress before and after divorce [95], and sensitivity analyses here and previously suggested that divorce-related trajectories are relatively specific to MD. Nevertheless, the validity of our proxy for MD could be further established by future research.

The trajectory of antidepressant use might also reflect a treatment effect, i.e., antidepressant use declines after divorce due, not to the passing of a crisis, but to successful antidepressant treatment of MD. Antidepressants have a large negative effect on depression symptoms, but placebos have an almost equally large negative effect [96]. Thus, if antidepressant use does reduce MD symptoms, reducing subsequent demand for antidepressants, this is largely a placebo effect, which is difficult to interpret under either mainstream illness models or adaptive models of MD. One evolutionary interpretation of the placebo effect is that if MD, in part, is a signal of need, then drug treatment (active or placebo) communicates to the patient that important members of society (doctors) take this need seriously, which might reduce need signalling (i.e., MD symptoms) [97,98]. Future modelling should incorporate direct placebo and active effects of treatment on MD symptoms, including possible psychotherapy status.

In formulating our adaptive model, we make some simplifying assumptions. For example, divorce has a bilateral (game-theoretic) structure, but our model is a unilateral description of divorce. Some evolutionary models [45,47] and empirical findings [47] speak for the importance of game-theoretic interactions in depression, and future modelling extensions in this direction might turn out to be useful. As another example, in our model, retaining an established relationship always entails more reproductive success than divorce, but some people might actively switch partners in hope of better prospects. However, while our discussion is presented in terms of postponing divorce, essentially the same cost-structures may apply when the individual attempts to switch partners rapidly without losing both; thus, the model prediction is largely in line with multiple adaptive explanations from bargaining to more general social-problem solving.

Finally, one of the important but less studied assumptions of our evolutionary adaptive model was that MD provides benefits during adversity. While this critical assumption needs further empirical evidence, some does exist. For example, detailed studies of family interactions with, e.g., a depressed spouse, parent, or child have documented that despite the negative reaction of others to depressed individuals, depressive behaviours elicit benefits, such as helping, problem solving and reduced aggression [99–101], consistent with social subordination and signalling theories of MD. In addition, a survey of suicidal behaviour in the ethnographic record found that it often succeeded in influencing mating decisions if the victim survived, such as preventing unwanted marriages, achieving prohibited marriages, and deterring abandonment [46]. After adolescent suicide attempts, parents have been found to express more caring, sympathy and support [102]. Given the enormous financial incentives to suppress MD chemically, it is imperative that mental health researchers convincingly rule out any possible benefits of reactive depression. The above studies and our results suggest that such benefits might exist. In addition, our model estimation suggests that modern-day decrease in 3-month probability of divorce due to MD may be quite small, even if adaptive. Only 0.007 units decrease from the baseline probability of 0.07, or odds ratio 0.89 of divorce for those who respond using MD in comparison to those who do not.

### Summary

This paper found that it is the period before the divorce that exposes people to depression, not the period after it. Presumably this is because of the social conflicts that precede the divorce. Evolutionary, adaptive models of depression suggest that it is a mechanism for coping with adversity, such as marital conflicts; they link onset of MD with the onset of adversity. We found that such a mechanistic model of adaptive behaviour was the best explanation for the observed temporal pattern of antidepressant purchases around the time of divorce among the four studied models. The evolutionary adaptive model also reproduced curvature properties of the data better than the other models. The simple stress-relief was also a very good fit to the data, numerically superseding the adaptive model when studying SSRI antidepressants only, but it provides no theoretical explanation for the specific time of MD onset or individual differences in the onset. The mechanistic adaptive model provides a solid basis for further scientific study, and our study also encourages development of mechanistically informed stress-relief models.

## Supporting information

S1 Text Sensitivity analyses and demographic parameterization. (PDF)

S1 Dataset Prevalence data used in model estimation. (CSV)

S1 Code R-language script providing an example of model estimation. (R)
