# Supplementary material for: Adaptive and non-adaptive models of depression: A comparison using register data on antidepressant medication during divorce
Source: PLoS One. 2017 Jun 14;12(6):e0179495. doi: 10.1371/journal.pone.0179495 (PMC5470737; doi:10.1371/journal.pone.0179495)
Supplement: S1 Text — (PDF) [file pone.0179495.s001.pdf]

Supplementary material

Adaptive and non-adaptive models of depression: a comparison using register data on antidepressant medication during divorce

Rosenström, T.[1,2,*]

Fawcett, T. W.[1,3]

Higginson, A. D.[1,3]

Metsä-Simola, N.[4]

Hagen, E. H.[5]

Houston, A. I.[1]

Martikainen, P.[4]

[1]School of Biological Sciences, University of Bristol, United Kingdom

[2]Institute of Behavioural Sciences, University of Helsinki, Finland

[3]Centre for Research in Animal Behaviour, University of Exeter, United Kingdom

[4]Population Research Unit, Department of Social Research, University of Helsinki, Finland

[5]Department of Anthropology, Washington State University, Vancouver, USA

[*]Correspondence: tom.rosenstrom@helsinki.fi

In the main manuscript we used a relatively simply version of the evolutionary adaptive model, which is in line with the general notation that “models should be as simple as possible but no simpler”. Although there are compelling reasons to avoid exact modern-day parametrization of the selective environments for behavioural states whose point of origin is unclear (see main text), such parametrization nevertheless serves as a good way to study robustness of the results. At least we know that the modern-day parameters are something that can happen in reality. This supplementary text present a more complex version of evolutionary state dependent model that uses a modern-day parametrization for the studied population. The simpler model in the main manuscript is nested within (a restricted version) of the more complex model; for brevity, we also discuss general aspects of both models in this text. We call the larger model a demographical parameterization of the optimal-behaviour model.

Demographically parameterized model

The demographical parameterization was based on the data released by Statistics Finland [1–3], and on previous studies using their data [4,5]. Below Figure summarizes these data as they were used. Mortality rate was well described by fitted constant, linear, and quadratic terms (cubic term no longer improved the fit: $p > 0.5$), and the model prediction was used rather than the somewhat idiosyncratic raw estimates (see panel A for the data and model predictions). Similarly, a convenient parametrization was chosen for fertility and marriage rates so as to approximate the published graphs under visual inspection (panels B–C).[1] Figure S1D shows that the fit of the demographically

1 Specifically, the fertility-rate function for men was given by the density function of the log-normal distribution (location 0 and scale ½) between values 0.3 and 2.0 spread to the interval of ages 20 to 40 by an affine transformation, and re-scaled so that the maximum annual fertility approximately corresponded to the observed rate (150 births per 1000 persons). Female fertility was otherwise the same, but with the scale parameter 0.4 and a 3-year translation of the x-axis, to reflect the earlier reproductive period compared to men. Female marriage rate was also log-normal on the interval 0.4 to 1.6 with scale 0.4 (males ‘delayed’ by two years). In addition to noise reduction, the parametric representation was useful for the below-described sensitivity analysis (Figure S2E–F) with respect to age-patterns, where we extrapolated mortality, fertility and marrying rates to older age groups.

parameterized model was nearly as good as for the simple model. The slightly lower coefficients of determination than for the simple model may be due to decreasing fertility at the old age. Overall, however, we took the good fit of both the simple and the demographically parameterized model to indicate robustness of the main results under different conditions. We also performed further sensitivity analyses on the demographically parameterized model, as described below. Before the results, the definition of the demographically parameterized model is given.

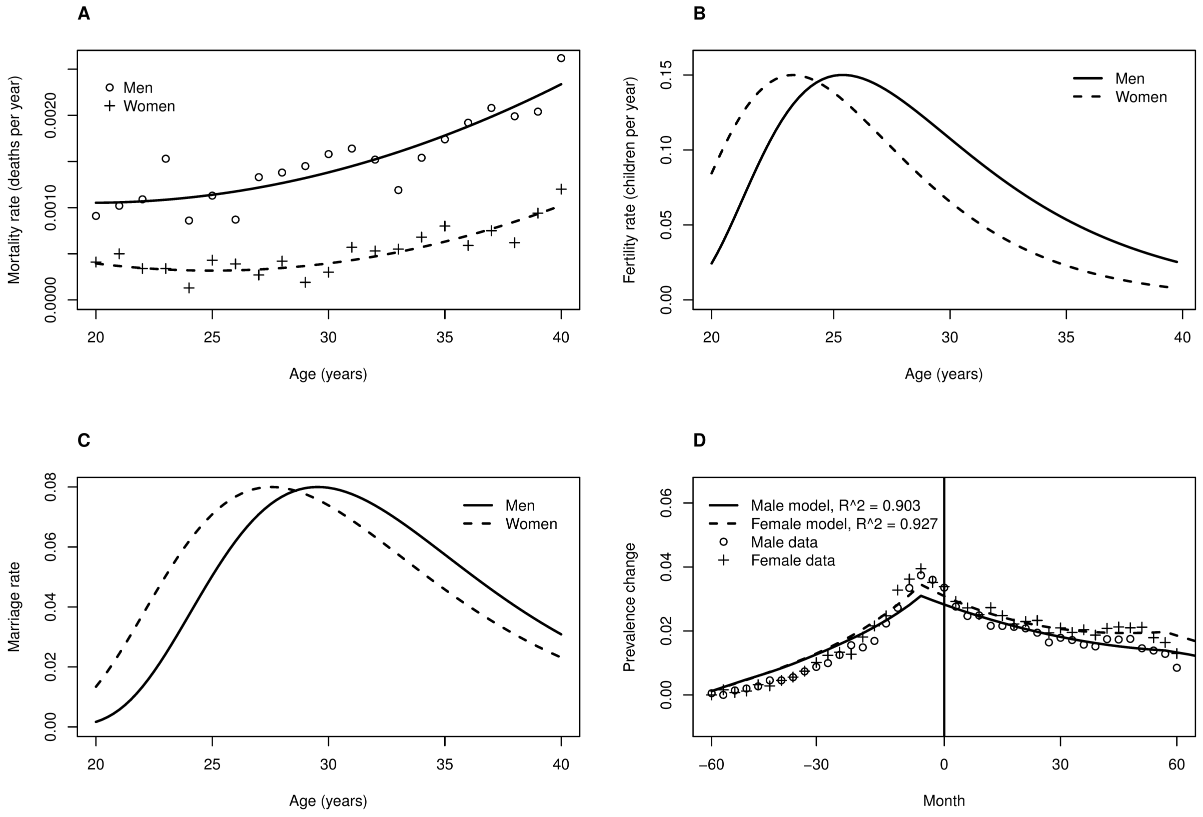

Supplementary Figure 1. Demographic data and the fit of the state-dependent optimality model in the implied 'realistic' environment. The panels show age-dependent (A) *mortality rate*, (B) *rate of offspring production, and* (C) *marrying rate, whereas panel* (D) *shows the optimality model fit in these age-dependent environments, separately for male and female data.*

Definition of the demographically parameterized model

The demographically parameterized model is defined by the below figure, which extends that of the main manuscript; furthermore, the below table provides the explicit representation in terms of transition matrices of probability theory.

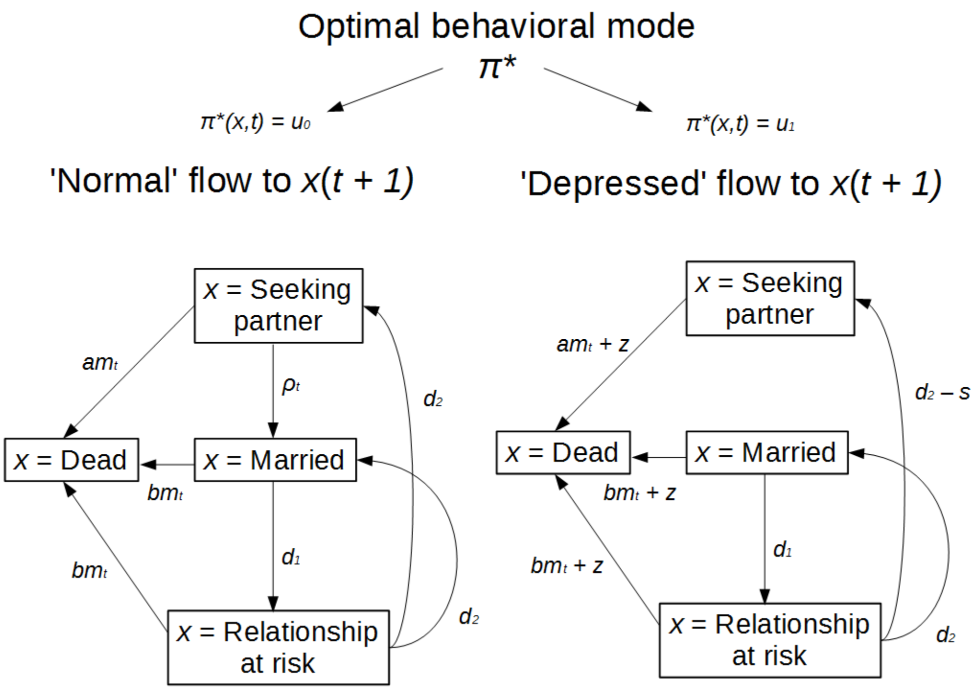

Supplementary Figure 2. Possible state transitions in the evolutionary state-dependent model. Note that it is also possible to stay in the same state for longer than one time step, but for clarity the self-loops are not shown (but see below Table). A 'strategy' $\pi(x,t)$ *defines whether to be in a 'depressed' mode* u_1 *or not (mode* u_0*) given the state* x *and time* t*. The star superscript refers specifically to the optimal strategy that maximizes the reproductive value. The choice of mode dictates the transition-probability structure for the next time step. The effect of the 'depressed' mode is to decrease the probability of a divorce-like transition from the relationship-at-risk state to the unpartnered state by the value* s*, to increase the (now possibly time- and state-dependent) probability of dying* am_t *(or* bm_t*) by* z*, and to remove the (possibly time-dependent) probability of marrying* ρ_t *(removal of* ρ_t *had no consequences here, but for some, it seems a logical outcome of 'depression'). For the demographically parameterized model, coefficients* a *and* b *reflected the fact the mortality rates are higher for unpartnered individuals* ($a > b$)*, but for the simpler model we assumed* $a = b = 1$*. We assumed that as many relationships at risk end up in divorce as in reconciliation on average* (d_2)*; the function* $f(d_1, d_2)$ *captured the overall divorce rate, as illustrated below.*

Table 1. The full stochastic state-transition matrices for the two behavioural modes, ‘normal’ (u_0) and ‘depressed’ (u_1), and the states ‘seeking partner’ (1), ‘married’ (2), ‘relationship at risk’ (3), and ‘dead’ (4)

State-transition matrix …				… when in mode u_0				… when in mode u_1			
p_{11}	p_{12}	p_{13}	p_{14}	$1 - am_t - \rho_t$	ρ_t	0	am_t	$1 - am_t - z$	0	0	$am_t + z$
p_{21}	p_{22}	p_{23}	p_{24}	0	$1 - d_1 - bm_t$	d_1	bm_t	0	$1 - z - d_1 - bm_t$	d_1	$bm_t + z$
p_{31}	p_{32}	p_{33}	p_{34}	d_2	d_2	$1 - 2d_2 - bm_t$	bm_t	$d_2 - s$	d_2	$1 + s - 2d_2 - z - bm_t$	$bm_t + z$
p_{41}	p_{42}	p_{43}	p_{44}	0	0	0	1	0	0	0	1

For any stochastic transition matrix the rows sum to one; that is, $\sum_j p_{ij} = 1$. State $x = 4$ (death) is absorbing (a zero probability of moving to other states). The now time-dependent parameters that were time-constant in the main manuscript we determined based on the above-described population data. The parameters a and b allow for incorporation of the empirical observation that mortality is higher in unmarried than in married individuals; specifically, being unmarried implies an approximately 2-fold mortality rate for men and 1.6-fold for women, despite adjustments for socio-economic position and household composition [4]. Thus, in the demographically parameterised model, $a = 4/3$ and $b = 2/3$ for men (i.e. $½am_t + ½bm_t = m_t$ and $a/b = 2$), whereas $a = 16/13$ and $b = 10/13$ for women. That is, this new model has slightly different and time-dependent parametrization compared to stationary model of the main manuscript; in all other respect, it was treated just the same. For both the model parameterizations, the following minor indeterminacy exists.

Going from state 2 (partnered, no relationship risk) to state 1 (unpartnered) takes two state transitions in our model, and the probability of making those within four 3-month periods (i.e. within a year) is given by

$$\sum_{k=0}^{2} p_{23}(1-p_{23})^k \sum_{t=0}^{3-k} p_{31}(1-p_{31})^{t-1} := f(p_{23}, p_{31}) \quad . \qquad \text{(S1)}$$

Thus, there are two parameters (p_{23} and p_{31}, mapped respectively to d_1 and d_2 in the mode u_0) and only one constraint (the annual divorce hazard). By examining the contours of f, illustrated in the below Figure, one observes that the convenient equal-importance choice of $p_{23} = d_1 = 0.07$ and $p_{31} = d_2 = 0.07$ yields an appropriate annual divorce hazard of ~0.026 [3]; the baseline values were thus set to 0.07 for the both parameters in the main paper. Herein, a sensitivity analysis then explored a range of values in the set $\{(d_1, d_2) \mid f(d_1, d_2) = 0.025\}$, as follows.

Sensitivity analyses

We explored values of d_1 in the range from 0.015 to 0.15, which implied a (declining) range from 0.359 to 0.031 for d_2 under the condition of a constant divorce rate (note that the transition structure of above Table limits the practically possible ranges in addition to the function f). Furthermore, we studied a range of selective-benefit values, s, from very small (0.001) to large (0.03). Supplementary Figure panels 3B and 3C show how the model fit (assessed by the coefficient of determination) for men and women changes as a function of these parameter values. The model fit is very robust against changes in d_1 and d_2, but if the benefit s from the mode u_l declines below a value of approximately 0.004, u_1 is no longer used and the model fit worsens, as one would expect. Nevertheless, there is a region for d_1 where the model fits particularly well ($R^2 > 0.9$); that is, for both sexes, the region $0.03 < d_l < 0.08$ yields a good fit, as long as s is sufficiently high (i.e., $s >$

0.004). This is the parameter regime where our model can explain the observed data reasonably well, and observing values outside of that range would constitute evidence against the model and its underlying hypotheses.

New qualitative insights may also arise from a systematic investigation of temporal changes in optimal behaviour, such as age-dependent patterns in reactions to divorce. We first studied how age and expected future reproductive value affected the choice of behavioural mode u_1 (depressed) in the simple model (dotted line in Supplementary Figure 3D). Considering a simulated population in which most people are initially (at age 20) unpartnered, they increasingly enter into marriage and then in some cases transition to the relationship-at-risk state in which u_1 is beneficial. This leads to a rapid initial rise in the general prevalence of u_1, followed by a long, relatively stable prevalence and a subsequent peak and drop in the last age periods. The peak towards the end reflects the fact that after the period the modelled reproductive resource 'ends'; keeping the resource is thus worth everything just before the final period and nothing in the exact final period. While conceptually relevant, these start- and end-game dynamics did not affect the divorce estimates in our simple constant-fertility model, because we excluded the first 20 and last 20 periods.

The simple model dealt more with the potential for reproduction (fecundity) than actual number of offspring (completed fertility), whereas our demographically parameterized model was set using data on completed fertility. In the latter model, the 'end effect' is much more pronounced due to a smoother, more rapid decline in the amount of completed fertility remaining (Supplementary Figure 3D). But there may be more at play than just end-period dynamics, because almost half the simulated population becomes depressed at the end. We thus roughly extrapolated the fertility, mortality, and partner-finding rates, simulating age periods 30–50, 40–60, and 50–70 years, in addition to the original 20–40 years. What happens is that completed male fertility gets very low and female fertility practically ends at the oldest age periods. Because the only currency that guides behaviour in our optimality model is the production of offspring, the use of the

behavioural mode u_1 around the time of divorce gradually becomes almost irrelevant for older men (Supplementary Figure 3E), and fully irrelevant for women after the menopause (Supplementary Figure 3F). This is of course a simplification: in reality, a behaviour that promotes the survival of offspring is still relevant for reproductive value, regardless of whether the focal individual has ceased reproducing. Nevertheless, our model reveals that theories based on maximizing direct offspring production predict a diminishing association between divorce and depressive reactions (and thus antidepressant purchases) as a function of the age at the time of divorce. This prediction was not backed up by the data, as shown in the Supplementary Figure 4.

Supplementary Figure 4 shows that divorce to antidepressant purchases link appears to strengthen by age. Other than the brief section in Discussion of the main manuscript, understanding this perplexing pattern is left for future studies A companion supplementary file provides the data points used in this work for possible further independent model development.

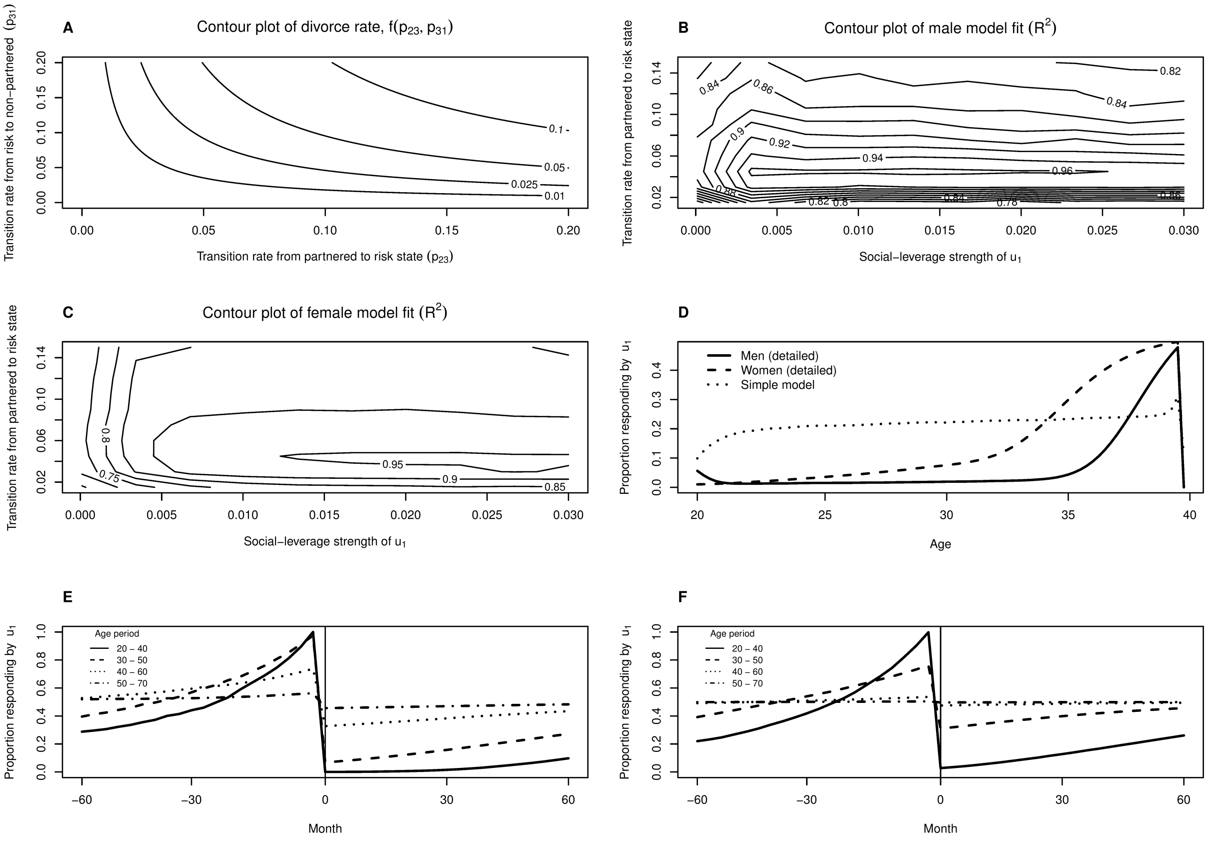

Supplementary Figure 3. Sensitivity analysis of model predictions. A) *A contour plot of annual divorce hazard resulting from a given 3-monthly rate of transition d_1 from partnered state (state 2) to relationship-at-risk state (state 3) versus the rate of transition d_2 from relationship-at-risk state to unpartnered state (state 1).* B) *A contour plot of the resulting model-to-data fit for a given value of the model parameter s (x-axis, representing gains from, or leverage of, the depressed mode (u_1) and a given value of the transition rate d_1 from partnered to risk state (y-axis; the transition d_2 is chosen so as to keep the annual divorce hazard $f(d_1, d_2)$ at the value 0.025). The other model details represent those of the demographically parameterized model.* C) *Same as panel* B *but for women.* D) *Proportion of simulated men in the 'depressed' mode u_1 for all the modelled time steps (x-axis) of the male (solid line) and female (dashed line) model and the simple constant-fertility model (dotted line).* E) *Proportion of men in the 'depressed' mode u_1 for each divorce-centred time point (i.e. underlying optimality-model prediction) in the simulated demographically realistic model. The different curves yield predictions for the different age groups (i.e. for divorce events occurring between 20 and 40 years, 30 and 50 years, and so on).* F) *Same as panel E but for women.*

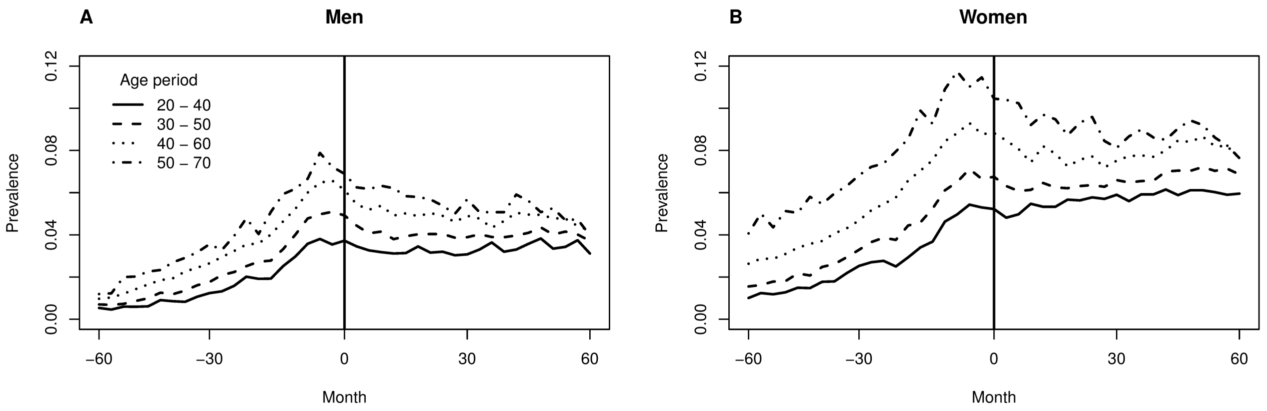

Supplementary Figure 4. Prevalence of antidepressant purchases around the time of first divorce for different age groups. The patterns were similar for the last divorces in the data (not shown). A) *Men with first divorce occurring between ages 20 and 40 (solid line), between ages 30 and 50 (dashed line), and so on.* B) *Same as panel A but for women.*

References

1. Statistics Finland 2008 Official Statistics of Finland (OSF): Mortality [online publication]. ISSN=1798-2529 [referred: 23.2.2015].

2. Statistics Finland 2009 Official Statistics of Finland (OSF): Population structure [e-publication]. ISSN=1797-5395. 2009, Population by age, marital status and sex 31.12.2009 [referred: 23.2.2015].

3. Statistics Finland 2013 Official Statistics of Finland (OSF): Changes in marital status [e-publication]. ISSN=1797-643X. 2012, Appendix figure 3. Divorce rate by age 1990, 2000, 2011 and 2012 [referred: 23.2.2015].

4. Martikainen, P., Martelin, T., Nihtilä, E., Majamaa, K. & Koskinen, S. 2005 Differences in mortality by marital status in Finland from 1976 to 2000: analyses of changes in marital-status distributions, socio-demographic and household composition, and cause of death. *Popul. Stud.* **59**, 99–115. (doi:10.1080/0032472052000332737)

5. Nisén, J., Martikainen, P., Silventoinen, K. & Myrskylä, M. 2014 Age-specific fertility by educational level in the Finnish male cohort born 1940–1950. *Demogr. Res.* **31**, 119–136. (doi:10.4054/DemRes.2014.31.5)
